# Effectiveness of family support-based companion video sharing to improve depression in perinatal maternity: a randomized controlled trial study protocol

**DOI:** 10.3389/fpsyt.2025.1641154

**Published:** 2025-10-22

**Authors:** Rantong Bao, Chenxin Yang, Ruijin Zhu, Wenzhuo Li, Yang Bai, Yang Jiang, Guoying Zhang

**Affiliations:** ^1^ Center for Clinical Epidemiology Research, The Affiliated Hospital of Inner Mongolia Medical University, Hohhot, China; ^2^ School of Health Management, China Medical University, Shenyang, China; ^3^ School of Nursing, Henan University of Science and Technology, Luoyang, China; ^4^ Medical College, Qingdao University, Qingdao, China; ^5^ School of Population and Health, Renmin University of China, Beijing, China; ^6^ Jitang College, North China University of Science and Technology, Tangshan, China; ^7^ Obstetrical Department, Dongying People’s Hospital, Dongying, China

**Keywords:** perinatal, family-based mHealth intervention, companion video sharing, postpartum depression, mindfulness intervention

## Abstract

**Background:**

The importance of family support in addressing maternal anxiety and depression during pregnancy is widely recognized. However, cultural nuances and healthcare system dynamics in China call for tailored interventions for perinatal mental health. Family-based companion video sharing via mobile health (mHealth) emerges as a potentially effective and scalable approach, delivering cost-effective emotional support and information dissemination. It is imperative to conduct rigorous evaluations through randomized controlled trials to assess its impact on maternal mental health. This study aims to evaluate the efficacy of family support-based mobile companion video sharing, providing data support for advancing perinatal mental health interventions in China.

**Methods:**

The study involves 40 pregnant women with mild to moderate depression symptoms and conducts an eight-month randomized controlled trial. Participants are randomly assigned to the intervention or control group. The intervention group receives a mHealth intervention involving six weeks of themed video sharing with their caregivers, based on mindfulness theory. Participants are required to record and share videos with their caregivers via WeChat according to weekly themes, in collaboration with the research team. The research team also regularly sends healthcare messages, creating a bidirectional intervention. The control group only receives healthcare messages. All participants are required to complete five follow-up visits, with depression levels assessed using the 5-item short form of the EPDS (EPDS-Dep-5).

**Discussion:**

This study innovatively explores mHealth interventions, specifically family-based companion video sharing to improve maternal mental health during pregnancy. In contrast to traditional interventions, this study emphasizes two-way communication between the mother and her companion, facilitating mutual support. If successful, this approach could inform perinatal mental health interventions globally.

**Trial registration:**

This study registered at ClinicalTrials.gov (ChiCTR2400084685) on May 22, 2024.

## 1 Background

The shift from the traditional biomedical model to the biopsychosocial framework has increased focus on mental health issues (1). The implementation of China’s two-child and three-child policies, leading to more pregnant women, has drawn attention to the mental health of perinatal women (2). Perinatal depression (PND), characterized by symptoms such as despondency and anxiety during pregnancy, childbirth, or after abortion, is a common complication in both gestational and postpartum periods (3, 4). Factors such as social support, occupational factors, healthcare access, and fluctuations in maternal hormone levels can predispose pregnant women to anxiety and depression (5–7). PND exerts deleterious effects not only on the mother and her immediate family but also has negative impacts on the physical and psychological well-being of the child, leading to outcomes like preterm labor, mother-infant bonding, and low birth weight (8–11). The prevalence of antenatal depression ranges from 15.0% to 25.0%, while postnatal depression incidence varies between 7.0% and 30.0%. In China, the overall prevalence of perinatal depression is estimated at approximately 16.3% (12, 13). A discernible correlation exists between the prevalence of PND and a nation’s level of development, with higher-income countries exhibiting lower prevalence rates compared to middle- and low-income counterparts (14). The prevalence of maternal depression varies across different life stages, with the cumulative prevalence rates for perinatal depression, antenatal depression, and postpartum depression averaging at 26.3%, 28.5%, and 27.6%, respectively (15). A study conducted in the United States revealed that nearly 50% of perinatal pregnant women displaying major depressive symptoms did not initiate timely treatment, with over 75% failing to adhere to treatment regimens (16). During the COVID-19 pandemic, the prevalence of prenatal depression in China surged to 71.0%, while in Poland, the prevalence of postpartum depression approached 74.0% (17). In recent years, effective prevention and management strategies for PND have emerged as crucial challenges receiving attention from scholars worldwide.

Medication-based treatments for depression have detrimental effects on both maternal and offspring health. Therefore, targeting psychosocial factors in perinatal depression (PND) shows promise as an effective strategy. Traditional interventions, including nursing protocols, health promotion, and psychological therapies such as interpersonal psychotherapy (IPT) and relaxation techniques, aim to alleviate perinatal anxiety and depression (18–22). These interventions often last weeks to months and face challenges with patient adherence, which can limit their effectiveness in reducing PND. Thus, it is urgently necessary to explore novel approaches to improve treatment outcomes (23).

Mobile Health (mHealth) has garnered attention as a promising avenue, with the World Health Organization (WHO) actively promotes adopting digital technologies to improve various patient care models (24–28). mHealth apps play a pivotal role in patient education, disease self-management, remote patient monitoring, and overall improvement of quality of life (29–31). For instance, the Interactive Maternal Information and Emotional Support Group (IMAGINE) demonstrated improved emotional management and acceptance of mental health care. This was achieved through personalized communication with guidance specialists and peer support (32). Similarly, a Japanese mHealth-based randomized controlled trial (RCT) showcased the preventive impact of a mobile health counseling app on postpartum depressive symptoms (33). Studies on Positive Thinking-Based Interventions (MBIs) have highlighted the efficacy of digitally guided self-help interventions in alleviating maternal depression throughout the perinatal period, with mHealth-based interventions exhibiting lower dropout rates and greater acceptability compared to face-to-face interventions (34–36). These interventions use various mHealth platforms to deliver support, such as online support groups, email or text messaging, mobile apps, and web pages (32, 33, 37–40). Despite the widespread use of mHealth, its effectiveness in reducing perinatal depression or anxiety remains inconclusive, with only a few apps being considered high quality (41, 42). Therefore, there is a critical need for the development of high-quality mHealth apps and rigorous evaluation of their potential to improve perinatal maternal mental health. This field warrants further research and investment to provide effective and accessible support for pregnant women experiencing depression and anxiety.

Studies have underscored the significant role of emotional, material, and informational support from family members in reducing maternal anxiety and depression, thus promoting healthy mental well-being during pregnancy (43, 44). Moreover, community-based comprehensive family interventions, involving family members in the care of pregnant women with postpartum depression, have shown promise in alleviating depressive symptoms and improving overall mental health outcomes (45). Notably, Liu’s study emphasizes that engaging caregivers in the care process not only helps dispel misconceptions about depression but also enhances their understanding of proper daily care practices (46). In many countries, maternal support during pregnancy primarily involves hospitals and communities in tandem with routine care. However, in Chinese cities, it often relies heavily on hospital support alongside family involvement. Due to these cultural and healthcare differences, the impact of home-based maternal support on perinatal depression deserves special attention in China. One promising intervention is family-based companion video sharing, which can serve as an accessible mHealth strategy. Through sharing videos documenting the caregiving process, the mother’s daily experiences, and insights, emotional support and essential information can be swiftly delivered, meeting the mother’s care needs effectively. This intervention aligns with preferences for easily implementable and cost-effective solutions while addressing societal needs. Consequently, an urgent need exists for a randomized controlled trial (RCT) to evaluate the feasibility and long-term behavioral effects (The situation of depressive symptoms in pregnant women 4 weeks after delivery) of family support-based video sharing. This trial should particularly focus on improving perinatal maternal mental health among pregnant women in China. Such research would shed light on the efficacy and practicality of this intervention within the Chinese healthcare landscape.

According to the Individual and Family Self-Management Theory (IFSMT) (47), self-management is viewed as a relatively complex process, and the theory consists of three dimensions: content, process, and outcome. The theory emphasizes improving individual and family self-management processes to achieve positive results, including behavioral changes, use of health and wellness services (proximal outcomes), and health status, quality of life, and healthcare expenditures (distal outcomes). Therefore, the family support-based companion video focuses on the pregnant woman and her family members to address perinatal maternal depression. By capturing perspectives from both the pregnant woman and her companion, the intervention aims to improve maternal depression and related health indicators. In addition, the content of the intervention shared in the companion video draws from Mindfulness-Based Interventions (MBIs), a form of psychotherapy that redirects attention to present moment experiences (48). Based on mindfulness theory, different themes guide pregnant women to objectively view the changes in their physical, psychological, and social environments during pregnancy and childbirth. While MBIs have demonstrated benefits for pregnant women internationally, these interventions are still at an early stage of development in China (49, 50).

This study designs a randomized controlled trial to evaluate the intervention effect of family support-based mobile companion video sharing on the mental health of perinatal women. Compared to previous mobile health intervention studies that used text or images as the intervention medium, this study has two innovative points: 1) The intervention medium is WeChat video, and the intervention mode is a bidirectional interaction between researchers and participants; 2) The intervention emphasizes the active participation of participants’ family members. This innovative intervention approach is expected to provide new perspectives and effective means for perinatal mental health management.

## 2 Methods

### 2.1 Objectives

This study aims to evaluate how effective a mobile health intervention using family support-based companion video sharing is in reducing perinatal maternal depressive symptoms. Additionally, the study also aims to evaluate the acceptability and feasibility of companion video sharing among pregnant women and their family members, and to explore factors influencing to this intervention.

### 2.2 Trial design

**Figure 1:**
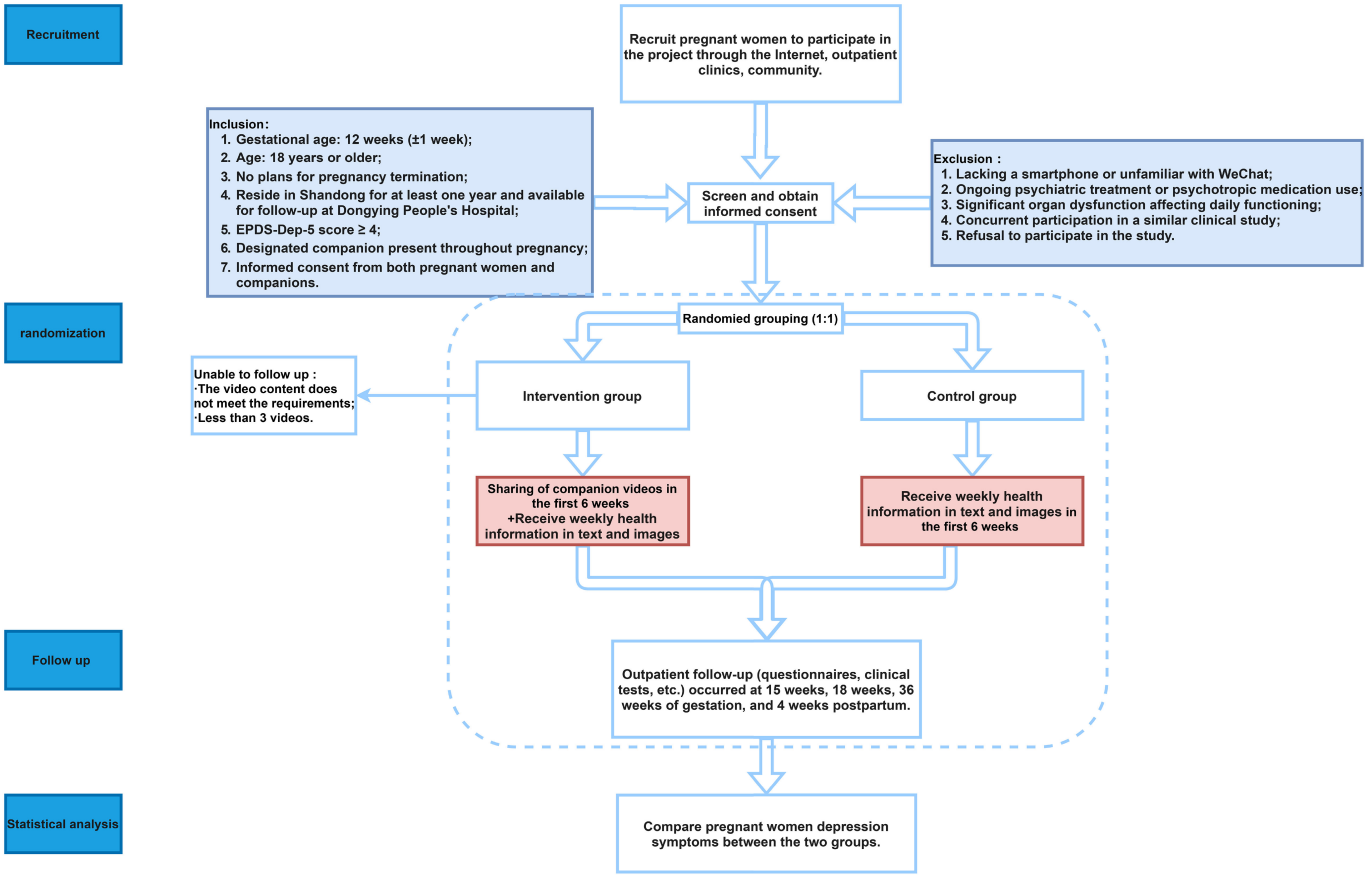
Study flowchart.

This study adopts a randomized controlled trial design and is conducted at Dongying People’s Hospital, Shandong Province, China. The intervention involves family support-based companion video sharing among perinatal women and their families. Forty participants are randomized into two groups: a 20-participant intervention group receiving companion video sharing and a 20-participant control group receiving traditional care. The research flowchart is shown in **Figure 1**.

### 2.3 Recruitment

We began recruiting perinatal pregnant women on August 12, 2024 using a multi-channel approach. This approach encompassed online platforms, hospital settings, and community outreach. Initially, announcements related to the trial were shared through the WeChat public account of Dongying People’s Hospital and its affiliated medical institutions, providing links to an electronic questionnaire for interested perinatal mothers and their companions to submit their contact information. Additionally, medical staff at the Obstetrics and Gynecology Specialist Outpatient Clinic of Dongying People’s Hospital inform perinatal pregnant women attending appointments about the study and provide questionnaires to those interested or showing signs of depression. Moreover, community health workers at various health service centers in urban areas associated with Dongying People’s Hospital promote the study and enroll eligible participants by administering questionnaires.

After identifying interested individuals who met the inclusion criteria, researchers clarified the study’s objectives and procedures. Then, the study’s medical personnel meticulously screened and selected eligible perinatal women based on predefined criteria. Each participant received a detailed letter of informed consent (refer to Appendix), detailing the study’s aims, procedures, and potential implications. After both parties mutually agreed and signed the informed consent, the participants who completed this process formed the recruited cohort. Random allocation then determined the assignment of participants to either the intervention group (companion video-sharing group) or the control group (traditional care group).

### 2.4 Eligibility criteria

#### 2.4.1 Inclusion criteria

(1) gestational age of 12 weeks (within a one-week window); (2) age of 18 years or older; (3) absence of plans for pregnancy termination; (4) commitment to reside in Shandong for at least one year and availability for study follow-up (with all visits conducted at Dongying People’s Hospital until delivery); (5) EPDS-Dep-5 score of ≥ 4 during depression screening (51); (6) consistent presence of a designated companion throughout pregnancy; and (7) comprehensive understanding and voluntary participation in the trial by both pregnant women and their companions (informed consent).

#### 2.4.2 Exclusion criteria

Exclusion criteria include: (1) lacking a smartphone or being unfamiliar with WeChat; (2) receiving ongoing psychiatric treatment or using psychotropic medications; (3) having significant organ dysfunction that impairs daily functioning; (4) participating concurrently in a similar depressive intervention clinical study; and (5) refusing to participate in the study.

### 2.5 Sample size

This study is a randomized controlled trial with an intervention group using companion video sharing and a control group receiving usual care. The primary outcome indicator observed is the EPDS score of the study population. Based on a previous intervention study (52), the EPDS score of the intervention group was 12.0 at 36 weeks’ gestation, while the control group had a score of 14.9 at the same gestational age. We set the hypothesis certainty (1-β) at 90%, and the significance level (α) at 0.05. Using PASS 15.0, the calculated sample size for the study was 32 participants, with 16 in the companion video sharing group and 16 in the traditional care group. Considering a 20% attrition rate, 40 participants were included in the study.

Group sample sizes of 20 and 20 achieve 99.472% power to reject the null hypothesis of equal means when the population mean difference is μ1 - μ2 = 12.0 - 14.9 = -2.9 with standard deviations of 2.4 for group 1 and 1.4 for group 2, and with a significance level (alpha) of 0.050 using a two-sided two-sample unequal-variance t-test.

### 2.6 Randomization

Following the baseline study visit, all participants were randomly assigned to one of the two study groups using a 1:1 randomization method. The randomization process involved using a random number table method.

Here’s the specific randomization process:

#### 2.6.1 Random number generation

A simple randomization method was used. The study designer generated a random number sequence using the RANDBETWEEN formula in Excel to create a sequence of numbers.

#### 2.6.2 Sealed envelopes

The sequences of numbers were kept in identical outer, sealed, opaque, numbered envelopes.

#### 2.6.3 Enrollment process

When each participant was enrolled, the researcher opened the envelopes sequentially according to a defined process.

#### 2.6.4 Assignment

The participant was then assigned to the corresponding group based on the number in the opened envelope, and the researcher made a record of the assignment results.

### 2.7 Interventions

The intervention content is grounded in mindfulness-based interventions (MBIs) and consists of six weekly themes designed to foster positive psychological states and healthy lifestyle routines among pregnant women. It integrates elements of the Individual and Family Self-Management Theory (IFSMT) and mobile-health strategies by leveraging the video-recording function of the WeChat app on smartphones: participants, accompanied by family members, record mindfulness videos on the assigned themes.

The multidisciplinary research team comprises one obstetrician, one obstetric nurse, and two research assistants. The obstetrician primarily handles maternal sudden illnesses and adverse reactions during pregnancy, while the obstetric nurse delivers positive thinking training tailored to specific intervention topics each week and addresses nursing and healthcare needs and precautions for pregnant women. The research assistants undertake various tasks, including registering study participant information, distributing and collecting questionnaires, disbursing subsidies. They also organize WeChat online video communications, downloading accompanying videos, and coordinating study follow-up visits.

The six-week Family Accompaniment Positive Thinking Intervention Program included six themes: 1) Understanding the Mindfulness-based Interventions Based on Family Accompaniment; 2) Speaking out about worries during pregnancy and childbirth; 3) How to better cope with adverse emotions during pregnancy and childbirth; 4) Familiarizing with the hospital’s maternity check-up process; 5) Each other in our eyes; and 6) Learning about maternal health. The specific intervention topics are detailed in **Table 1**.

**Table 1:** Detailed Description of Intervention Themes.

Item	Theme	Explanation	Content of Implementation	Feedback
1	Understanding the Mindfulnes-based Interventions Based on Family Accompaniment.	Let pregnant women understand what mindfulness training is; The reason to do mindfulness training; What mindfulness training can achieve; Why family care is needed and what family care can do.	Obstetric nurses explain the current domestic and foreign intervention methods for maternal depression to pregnant women. The obstetrical nurses introduce the content of Mindfulness-Based Interventions theory and individual & family self-management theory. Encourage pregnant women and their caregivers.	Send videos about “The Understanding of Companion video sharing based on Family- Support.”
2	Expressing Worries During Pregnancy and Childbirth.	Grounded in personalized-care theory, the causes of depression differ for each pregnant woman. By collecting the worries voiced by pregnant women and their companions, we develop targeted intervention strategies or provide individualized counseling to alleviate these concerns.	Guided by a nurse, the pregnant woman and her companion each express their worries about pregnancy and childbirth—for example, unfamiliarity with hospital procedures, lack of prenatal and postpartum knowledge, concerns about physical recovery, or fears about not being able to care for the baby adequately alongside family members. After worries are gathered, any existing coping or problem-solving strategies are shared through recorded videos.	Send videos about “Worries &Strategies”
3	How to cope with perinatal bad moods.	It is quite common for negative emotions to occur during pregnancy and childbirth; therefore, it is crucial to help women during the perinatal period master effective coping strategies to maintain their emotional well-being.	(1) Promote physical and mental activities: Pregnant and postpartum women should participate in appropriate physical activities, such as yoga and walking. (2) Maintain good sleep and dietary habits: Sleep and diet are vital for emotional well-being. It is recommended that women during the perinatal period maintain a regular routine and keep a balanced and nutritious diet. (3) Build a social support system: Establish a social support network for women during the perinatal period that includes family, friends, and colleagues, as their support and understanding are particularly critical for emotional management. Additionally, these strategies can significantly improve emotional health during this critical time.	Send videos about “One day vlog during pregnancy (exercise video / diet sharing / family companionship).”
4	Familiarize Yourself with the Hospital’s Prenatal Examination Process	Enhance pregnant women’s trust in the hospital, help them become familiar with the routine prenatal examination procedures, and improve the compliance of both the pregnant women and their companions.	(1) A nurse explains the hospital’s routine prenatal examination items and procedures. (2) The pregnant woman and her companion record a video titled “Hospital Prenatal Visit Walk-through.”	Send videos about “Hospital Prenatal Visit Walk-through.”
5	“The mutual perception in our eyes”	Grounded in family-centered support, the relationship between the pregnant woman and her partner (especially her husband) is pivotal. This session aims to help both parties recognize each other’s contributions during pregnancy, identify shortcomings, and resolve conflicts, laying the groundwork for the partners’ understanding and active participation in the intervention.	(1) A nurse explains the physical and emotional changes pregnant women experience. (2) The nurse guides the caregiver on how to collaborate for a safe and joyful pregnancy, highlighting common pitfalls such as insufficient partner support, criticism, and excessive maternal caloric intake leading to macrosomia.	Send videos about “Changes and Feelings About Each Other.”
6	Understanding Delivery	Help pregnant women recognize the delivery process, alleviate anxiety, enhance confidence, improve self-care awareness, actively participate in delivery, ensure maternal and infant safety, and make informed decisions to smoothly welcome the infant.	(1) Detailed introduction to the three stages of labor: cervical dilation stage, fetal delivery stage, and placental delivery stage, as well as possible situations and pain experiences in each stage; (2) Educational courses and lectures: Organize or recommend pregnant and puerperal women to participate in educational courses and lectures about the delivery process; (3) Watch educational videos: Provide educational videos about the delivery process for pregnant and puerperal women.	Send videos about "Understanding Delivery."

The intervention program comprised six themes, and each theme lasted a week and delivered via WeChat. It involved teaching on specific themes and filming videos. At the beginning of each week, the obstetric nurse presented the week’s theme content to the pregnant woman and her companion. She also provided relevant introductory materials, such as texts and videos. Pregnant women and their companions were asked to record a companion video related to family support for each theme and send it to the research group’s WeChat. Research assistants managed the videos to ensure they met the study criteria, and they will provide feedback when necessary. At the end of each week, there was a WeChat online video exchange where pregnant women and companions could ask the research team about perinatal clinical and nursing problems encountered during the week, and the team responded individually. Additionally, pregnant women completed a questionnaire via WeChat at each follow-up site.

The inclusion criteria for companion videos required both the pregnant woman and caregiver to appear in the video. They needed to describe and record content based on the weekly theme. Additionally, at least three companion videos had to be completed during the six-week intervention period, with each video filmed at intervals of one week or more. Exclusion criteria for companion videos included: repeated sharing of the same video; presence or speech by only one person; and videos shorter than one minute.

### 2.8 Adherence and monitoring

The study adopted a multi-pronged approach to improve compliance with the intervention protocol. First, a dedicated prenatal education program was established to provide participants with a comprehensive curriculum, ensuring they understood the objectives and procedures of the intervention. Second, participants in the intervention group were supported by a specialized research team; the project team regularly sent notifications and responded to questions from pregnant women. Third, participants were notified by SMS and telephone each intervention week to prompt them to engage with video intervention content on specified topics at predetermined times. Finally, we implemented six weeks of interventions on different topics based on common issues encountered during pregnancy and enhanced compliance by increasing the diversity of interventions and participants’ interest in participation.

### 2.9 Control group

Pregnant women in the control group receive perinatal health care materials prepared by obstetricians and obstetric nurses. These materials, provided weekly as text messages, images, or both via WeChat, are sent during the first six weeks after enrolment. Additionally, the research assistant sends reminders via WeChat to prompt them to complete questionnaires at each follow-up time point.

### 2.10 Outcomes evaluation

#### 2.10.1 Primary outcome

The severity of maternal anxiety and depression is primarily assessed by the Edinburgh Postnatal Depression Scale (EPDS), the scale was developed by Cox et al. (53) in 1987 and introduced in 1998. The revised EPDS consists of 10 items, each divided into 4 levels: “Never,” “Occasionally,” “Often,” and “Always,” corresponding to scores of 0, 1, 2, and 3, respectively, with items 1, 2 and 4 being reverse scored. The total score ranges from 0 to 30. The Cronbach’s alpha coefficient of this scale is 0.87, indicating high validity and reliability (54). The Chinese EPDS-Dep-5 scale used in this study consists of items 1, 2, 8, 9, and 10 from the EPDS scale, and the cutoff score for the EPDS-Dep-5 scale is 4 points, meaning that when the total score of the assessment is ≥4 points, it indicates that the pregnant woman is at high risk for depression. Although the full version of the EPDS is more commonly used, this study chose the EPDS-Dep-5 mainly based on the following considerations: (1) This 5-item short version, which has been shown to be highly correlated with the full version (r≈0.90), does a good job of distinguishing depression in terms of sensitivity and specificity; (2) The sample size of this study is relatively small, and repeated assessments during the intervention process require a shorter version to reduce participant burden and reduce the chances of losing participants; (3) The short version can be completed in just 0.5–1 minute, making it better for new moms in the early days when they might not have much energy.

#### 2.10.2 Secondary outcomes

##### 2.10.2.1 Social support and psychological well-being

The Chinese Generalized Anxiety Disorder Scale (CGAD)will be used to measure the severity and functional impact of maternal anxiety in the last two weeks. The scale was developed by Spitzer et al (55). The scale consists of seven mood entries. Each entry is categorized into four levels: 0 means not at all, 1 means several days, 2 means more than a week, and 3 means almost every day. The scale has a total score of 21 points. Scores of 0–4 indicate no anxiety; 5–9 indicate mild anxiety; 10–14 indicate moderate anxiety; and scores above 15 indicate severe anxiety. The higher the score, the higher the score, the higher the degree of anxiety. Spitzer et al. (55) found that in a large sample survey, when using a score of 10 as the cut-off point, it had a sensitivity and specificity of 89 per cent and 82 per cent, respectively. The scale has a high Cronbach alpha coefficient of 0.92, indicating strong reliability and validity.

The Chinese Perceived Stress Scale (CPSS) will be used to assess the degree of perceived stress in mothers who perceive their lives as unpredictable, uncontrollable, or overloaded. The scale is divided into 4 entries, each of which is classified into 5 levels, ranging from 0 = “never” to 4 = “very often”. For scoring, a Likert 5-point scale is used, with 2 entries scored as forward scoring questions (1, 4) and 2 entries scored as reverse scoring questions (2, 3). Higher scores indicate higher perceived stress. The Cronbach’s alpha coefficient for this scale is 0.754 (56).

Maternal sleep quality will be assessed using the Chinese Pittsburgh Sleep Quality Index (CPSQI), the scale developed by Buysse et al. (57) in 1989 to assess the patient’s sleep over the past 1 month. The 19 individual entries yielded seven “component” scores, each of which was divided into four levels: sleep quality, time to sleep, sleep duration, sleep efficiency, sleep disorders, hypnotic medications, and daytime dysfunction (58). Entries 1 to 4 are subjective questions. Entries 5a to 5j, 7, and 8 are rated on a four-level scale: “none” scores 0, “<1 time/week” scores 1, “1–2 times/week” scores 2, and “≥3 times/week” scores 3. The response score for entry 6 is 0 points for “very good”, 1 point for “good”, 2 points for “poor”, and 3 points for “very poor”. The response score for entry 9 is 0 for “No”, 1 for “Occasionally”, 2 for “Sometimes”, and 3 for “Often”. The total score of the scale is the sum of the seven “component” scores, which range from 0 to 21, with a higher total score implying poorer sleep quality. A total score of more than 5 is considered to be sleep disturbance; a total score of more than 7 indicates poor sleep quality (59, 60).

Patients’ fatigue severity will be assessed using the Chinese Fatigue Severity Scale (CFSS), which has 9 entries. A Likert 7-point scale is used, with 1 indicating strong disagreement, and 7 indicating strong agreement. The average of the scores for the 9 questions is used for scoring. An average score of ≥4 indicates symptoms of fatigue. The higher the total score, the greater the degree of fatigue. The Cronbach’s alpha coefficient of the scale is 0.81 to 0.89, which has good reliability and validity (61).

The Chinese Family Health Scale Short-Form (CFHS-SF) is used to measure maternal family health functioning. The scale consists of 4 dimensions and 10 items. The 4 dimensions include: family/social/emotional health processes, family healthy lifestyle, family health resources, and external social support for the family. Each entry is scored on a 5-point scale ranging from “strongly disagree” to “strongly agree” (on a scale of 1 to 5), with questions 6, 9, and 10 being reverse scored. The higher the total score, the better the level of maternal family health. The Cronbach’s alpha coefficient for this scale is 0.846 (62, 63).

The level of social support will be assessed by the Chinese Perceived Social Support Scale (CPSSS). The scale consists of 3 dimensions and 12 items. The 3 dimensions include family support (items 3, 4, 8, and 11), friend support (items 6, 7, 9, and 12), and other support (items 1, 2, 5, and 10), and each item is rated on a 7-point scale (1 to 7). For positive scoring, each item is rated on a 7-point scale (1 = “not at all compatible”, 7 = “fully compatible”). The higher the total score, the higher the level of social support. Scores from 12 to 36 indicate low support, 37 to 60 indicate intermediate support, and 61 to 84 indicate high support (64).The Cronbach’s alpha coefficient for this scale is 0.925, which has high validity and reliability (65).

The Chinese Big Five Personality Inventory Brief Version (CBF-PI-B) will be used to evaluate the effect of maternal personality. This inventory consists of 5 dimensions and 40 personality items. These dimensions are neuroticism, extraversion, openness, agreeableness, and conscientiousness. Each item is scored on a 6-point scale: 1 = not at all, 2 = mostly not, 3 = somewhat not, 4 = somewhat, 5 = mostly, and 6 = fully. Each dimension is treated as a separate subscale and each subscale is scored separately. High and low score thresholds for each trait are as follows: neuroticism ≥ 25.9 (high), ≤ 8.5 (low); extraversion ≥ 28 (high), ≤ 17.3 (low); openness ≥ 31.3 (high), ≤ 21.3 (low); agreeableness ≥ 32 (high), ≤ 20 (low); conscientiousness ≥ 29.3 (high), < 24 (low) (66). The scales showed good internal consistency, with an average coefficient of 0.793. Test-retest reliability over 10 weeks averaged 0.742, indicating high reliability and validity (67).

##### 2.10.2.2 Willingness to accept companion video sharing

This section assesses the types of smart devices that participants can operate independently, whether they have used smart devices for health monitoring, their acceptance of the concept of mHealth intervention strategies, and their self-assessed ability to use smart devices. It aims to identify factors hindering pregnant women and their companions from using smartphones to capture companion videos for health monitoring. These factors may include privacy concerns related to the videos, reluctance of companions to participate in video recording, and difficulties in operating the video recording process.

##### 2.10.2.3 Adherence to companion video sharing

Adherence to the companion video sharing intervention will be assessed based on the frequency and consistency of video sharing by each pregnant woman.

##### 2.10.2.4 Economic indicators

Costs incurred during clinic visits, including transportation, examinations, hospitalization, surgery, medication, nutrition, and lost work time.

This study will also collect comprehensive information from participants via an electronic questionnaire, including:

- Socio-demographic characteristics such as height, weight, age, education level, marital status, ethnicity, and income;
- Behavioral characteristics including smoking, drinking, disease history, eating, exercise, and sleeping habits;
- Maternal pregnancy indicators like pregnancy stage and prior pregnancy experience;
- History of depression diagnosis and treatment, including past antidepressant use and self-assessed health status;
- Experience and health knowledge of maternal companions, including previous companion experience and related health knowledge.

### 2.11 Follow-up

This study will last for eight months. All participants, both in the intervention and control groups, are required to complete five follow-up assessments: baseline, mid-intervention at 15 weeks of pregnancy, end of intervention at 18 weeks of pregnancy, 36 weeks of pregnancy, and 4 weeks postpartum. The content of these follow-up assessments varies according to the time point. Demographic information is collected only at baseline; behavioral data, clinical test results (such as blood pressure and glucose levels), and questionnaire responses are collected at all follow-up points. Specific details of the information collected at each follow-up are shown in **Table 2**.

**Table 2:** Follow-up Study Table.

Study period	Recruitment	Baseline	Intervention			
Timepoint		0w±1w (T1)	3w±1w (T2)	6w±1w (T3)	24w±1w (T4)	After delivery 4w±1w (T5)
Informed consent	✓					
Random grouping		✓				
Companion video sharing intervention		✓	✓	✓		
Send health-related text or images		✓	✓	✓		
Sociodemographic information		✓				
Behavioral information		✓	✓	✓	✓	✓
Medical history, physical examination (blood pressure, body height, weight, waist circumference), laboratory examinations (blood biochemistry, routine urine test).		✓	✓	✓	✓	✓
EPDS-Dep-5	✓	✓	✓	✓	✓	✓
GAD-7		✓	✓	✓	✓	✓
PSS		✓	✓	✓	✓	✓
PSQI		✓	✓	✓	✓	✓
FSS		✓	✓	✓	✓	✓
FHS-SF		✓	✓	✓	✓	✓
PSSS		✓	✓	✓	✓	✓
CBF-PI-B		✓				

The full name of EPDS-Dep-5 is the simplified version of the Edinburgh Postnatal Depression Scale; The full name of GAD-7 is Generalized Anxiety Disorder scale; The full name of PPS is Perceived Stress Scale; The full name is PSQI is Pittsburgh Sleep Quality Index; The full name of FSS is Fatigue Severity Scale; The full name of FHS-SF is The Family Health Scale short-form; The full name of PSSS is Perceived Social Support Scale; The full name of CBF-PI-B is The Chinese Big Five Personality Inventory Brief Version.

### 2.12 Statistical methods

Upon completion of the trial, all collected case data and related questionnaire responses were collated and summarized. Microsoft Office 2016 was utilized for data storage; statistical analysis was conducted using SPSS Statistics 27.0 software. Count data were presented as both the number of cases (N) and percentage (%). Continuous data distribution was assessed for normality. Normally distributed data were expressed as mean ± standard deviation, whereas non-normally distributed data were presented as median (IQR, P25–P75). Baseline characteristics between the intervention and control groups were compared and analyzed using appropriate statistical tests. The choice of tests—including two independent samples t-test, Mann-Whitney U-test, and chi-square test—depended on the nature of the data. Longitudinal analysis of follow-up data was conducted using Generalized Estimating Equations (GEE) to compare differences in indicators between the intervention and control groups. A two-sided test was employed, with statistical significance set at the *P* < 0.05.

Regarding missing data, multiple imputation techniques were employed, justified by the missing at random (MAR) nature of the missing data. Multiple imputation involves generating multiple filled datasets by replacing missing values with reasonable estimates derived from observed data and imputation models. This method reflects the uncertainty of missing data, maintains dataset variability, and enhances result reliability compared to single imputation methods. Multiple imputation methods were implemented using established statistical packages such as SPSS or R. Sensitivity analyses were also performed to assess result robustness under various missing data assumptions. Therefore, this comprehensive approach ensures the validity and reliability of the study findings, accounting for missing data while maintaining statistical integrity throughout the analysis process.

### 2.13 Study management

#### 2.13.1 Data collection

All study participants will complete corresponding questionnaires at baseline and during follow-up visits. The Questionnaire Star platform will provide the online questionnaires via a QR code, which participants will scan to complete them. Additionally, pregnant women sociodemographic and behavioral information will be collected using the same method through the case system and self-designed questionnaires.

#### 2.13.2 Storage and archiving of data

The investigators stored all research-related data (e.g., baseline and follow-up questionnaires, randomization records, follow-up information lists) and documents (e.g., subject informed consent forms, shared accompanying video files) in a standardized format within a dedicated research database. Following trial completion, all source data and documents were archived in compliance with applicable laws and regulations. Access to the database required investigator permission, with mandatory registration of visitor details and access purpose to prevent unauthorized access and data misuse (68).

#### 2.13.3 Confidentiality

To ensure participant privacy, this study will adhere to the *Personal Information Protection Law of the People’s Republic of China* and relevant regulations, implementing the following measures:

##### 2.13.3.1 Categorized and hierarchical management

Participant identifiers (e.g., names, ID numbers), health information, and contact details will undergo classified hierarchical management with labeled organization. This ensures data of varying sensitivity levels receive commensurate security protection.

##### 2.13.3.2 Encrypted data transmission

Encryption protocols will be implemented during data transmission. Decryption keys will be securely transmitted to recipients through separate, authenticated channels.

##### 2.13.3.3 Encrypted storage

Personal information will be stored in encrypted form, adhering to national cryptographic management standards.

##### 2.13.3.4 Segregation of biometric data

Where feasible, biometric information (e.g., fingerprints, facial recognition data) will be stored separately from personally identifiable information. Unless strictly necessary, raw biometric data will not be retained; only *cryptographic digest information* will be preserved.

### 2.14 Investigator responsibilities

Researchers hold important responsibilities in clinical trials, including ensuring compliance with ethical and scientific standards, protecting subjects’ rights, maintaining data integrity, overseeing trial implementation, and promptly reporting adverse events. Additionally, all participating team members must undergo regular training and supervision to ensure strict adherence to the study protocol and relevant regulations, and to standardize and accurately collect data.

### 2.15 Approval of trial protocol and amendments

This study will follow the *Ethical Review Measures for Biomedical Research Involving Human Subjects* and the ethical review and clinical trial management regulations issued by the National Health Commission. The trial protocol and informed consent form will be submitted to the Ethics Committee for review before the start of the trial, and require written approval. Any modifications to the trial protocol, including key changes in research design, subject population, sample size, etc., must be submitted in writing to the Ethics Committee before implementation. In urgent situations where deviations are necessary to eliminate an immediate hazard to subjects, the researcher may act first but must immediately report the deviation with justification to both the Ethics Committee and the sponsor. All protocol modifications and approval records will be securely archived to ensure trial transparency and regulatory compliance.

### 2.16 Data monitoring

A Data Monitoring Committee (DMC) will be established for this study, consisting of at least 2 members of the Medical Ethics Committee of Dongying City People’s Hospital. The DMC will work according to a pre-established Data Monitoring Plan (DMP) and will be responsible for periodic assessment of the progress and safety data of the trial. Functioning independently and separately from the research team, its primary responsibilities include monitoring data integrity, evaluating subject safety, and safeguarding participant rights. All monitoring activities were performed in a blinded manner to ensure objectivity of data and reliability of trial results. If any non-compliance with the approved study protocol or any unauthorized changes in study procedures are discovered, the DMC has the right to immediately suspend or terminate the study. The final trial dataset will be accessible to the principal investigator, co-investigators, and sponsor-designated personnel, while also remaining available for review by the DMC and external auditors.

### 2.17 Safety

Adverse events (AEs) in pregnant participants will be monitored throughout the intervention period, including but not limited to psychological reactions, mood fluctuations, behavioral changes, and difficulties with technology usage. All AEs will be recorded in detail, encompassing severity, time of onset, duration, interventions applied, and final outcomes. Any serious adverse events (SAEs) identified will be reported in accordance with the regulations and time requirements of the Medical Ethics Committee of Dongying People’s Hospital in Shandong Province. For urgent SAEs, the research team will immediately take necessary medical measures and promptly notify the Ethics Committee and the sponsor. The study will continuously evaluate the safety and applicability of interventions to ensure maternal safety and well-being, thereby generating critical safety data for the clinical trial.

## 3 Discussion

Currently, interventions for perinatal maternal depression can be divided into preventive and therapeutic interventions. Preventive interventions primarily focus on psychological interventions and are implemented for pregnant women showing signs of depression or at risk of depression; for those diagnosed with depression, a combination of drug and psychological therapy is used (69). Several studies have shown that drug therapy may have adverse effect on the neurological, behavioral, cognitive and emotional development of the fetus (70, 71). Thus, psychological interventions play a crucial role in the prevention and treatment of postpartum depression (72). Commonly supported theoretical interventions for maternal depression include positive thinking interventions (mindfulness-based interventions, MBI), cognitive behavioral therapy (CBT), and interpersonal therapy (IPT). Intervention channels based on mobile health, such as WeChat groups, hospital WeChat public accounts, and specially developed applications or websites, have become important platforms for communication between pregnant women and maternal and child health personnel (73). With these platforms, researchers can follow up with pregnant women, providing them with maternal and child health information during pregnancy and childbirth. This proactive approach has shown promise in preventing postpartum depression. However, most of the interventions are passively received by the mothers, with limited active feedback or output from them. In some cases, pregnant women’ feedback is limited to simple actions such as clicking links or filling out questionnaires, making it difficult to fully support pregnant women with depressive tendencies in expressing their emotions, thus limiting the effectiveness of interventions in improving maternal depression and enhancing their acceptance. A meta-analysis demonstrated the short-term efficacy of mindfulness-based interventions (MBI) during pregnancy for postpartum depression (PPD), but long-term outcomes remain unclear (74).

Compared with other similar interventions that include family support, this study focuses on a new, personalized behavioral intervention to reduce the risk of depression occurrence and further deterioration among pregnant women in China. The innovation of this mobile device-based intervention lies in retaining traditional nursing interventions, such as regularly delivering perinatal health knowledge to pregnant women, while also requiring pregnant women and their companions to actively record and share short videos of their experiences, thereby achieving a bidirectional interactive intervention. This intervention not only strengthens the effect of reducing depression symptoms among pregnant and puerperal women through companion-supported nursing but also shares records of different intervention themes in video form. This enhances feedback from pregnant and puerperal women to the research team and promotes interaction between them, thereby ensuring the effectiveness of the intervention. Therefore, family-supported companion video sharing may become a more effective strategy for alleviating depression among pregnant and puerperal women. This novel approach can provide preliminary evidence for the reduction of adverse psychological symptoms, such as depression, among pregnant women, as well as the long-term effects of such interventions.

This study involves the use of smart devices and the cooperation of caregivers. The research team believes that pregnant women may encounter the following situations when facing new interventions: 1) low acceptance of emerging things (such as mobile health strategies and video sharing formats); 2) concerns about personal privacy leakage, as the video content involves the recent lives of pregnant women and their partners; 3) insufficient cooperation from caregivers, as some caregivers may prefer traditional intervention and care models due to a lack of knowledge and experience related to accompanying pregnant women, leading to concerns about new interventions. In response to the above situations, the research team will present the latest research results on improving maternal depression through mobile health strategies to pregnant women and their caregivers during the initial recruitment phase, and explain that this study provides free professional clinical assessment and consultation services to pregnant women throughout the entire pregnancy. Pregnant women and caregivers who are screened will receive a one-on-one explanation of the requirements for video shooting during the start of the study to ensure the quality of the accompanying videos. Pregnant women and caregivers will be informed that the videos are managed by dedicated research assistants from the research team and that privacy will not be leaked, thereby alleviating their concerns. Additionally, there are a few limitations to this study, such as a small sample size, a single-center study, and variations in caregiver participation levels. Furthermore, this study only follows up to four weeks postpartum, which makes it hard to see the long-term effects on postpartum depression. To tackle the issues with how widely the results can be applied due to the small sample size and single-center trial, specific details about the intervention have been provided as **Supplementary Material** to ensure the reproducibility of the intervention measures. Future research is recommended to extend the follow-up period to look into how the intervention affects postpartum depression over time.

The results of this study will provide scientific evidence advocating for wider promotion and application of mobile health interventions to prevent and address health issues of pregnant and postpartum women during the peripartum period. By demonstrating the benefits of family support–based mobile companion video sharing—that is, sharing supportive videos via mobile devices among family members—this study may serve as a reference for medical policies and practices. This, in turn, can ultimately improve maternal care and outcomes. Moreover, this research is expected to pave the way for further studies and innovations utilizing digital technology to support the health of pregnant and postpartum women. It also encourages interdisciplinary collaboration among healthcare providers, technology experts, and policymakers, to optimize peripartum care services. Overall, investing in mobile health intervention research for managing peripartum mental health has great potential to enhance the well-being of pregnant women and promote maternal and infant health outcomes.
