# Supplementary material for: Effectiveness of family support-based companion video sharing to improve depression in perinatal maternity: a randomized controlled trial study protocol
Source: Front Psychiatry. 2025 Oct 22;16:1641154. doi: 10.3389/fpsyt.2025.1641154 (PMC12586068; doi:10.3389/fpsyt.2025.1641154)
Supplement: Supplementary file 1 [file Supplementaryfile1.docx]

**Informed Consent**

**Part I. Information for Subjects**

We are going to conduct a study called "Intervention Effectiveness of Family Support-Based Companion Video Sharing on Improvement of Perinatal Maternal Depression." You are eligible for enrollment in this study; we would like to invite you to participate. This informed consent form will inform you about the purpose, steps, benefits, risks, and possible inconvenience or discomfort you may experience as a result of this study, so please read it carefully and make an informed decision about whether or not to participate. As the researcher explains and discusses the informed consent form with you, you may ask questions at any time, and let him/her explain it to you. Any part you don't understand, you can ask questions. You can also discuss this with family, friends, and your attending doctor before making a decision. If you are currently participating in another clinical study, be sure to tell your research physician or investigator.

1. **Background and objective of the study:**

In recent years, the gradual implementation of the national two-child and three-child policies has led to an increase in the number of pregnant women in China, and greater attention has been given to maternal mental health during the perinatal period. In China, the prevalence of perinatal depression (PND) is approximately 16.3%, and studies have shown that the prevalence of prenatal depression reached as high as 71.0% during the COVID-19 pandemic. Traditional intervention programs aimed at reducing the risk of adverse psychological events, such as perinatal depression and anxiety, have limited effectiveness due to their infrequent sessions—spaced weeks to months apart—and challenges with patient adherence. Meanwhile, the rapid growth of mobile health (mHealth) technologies has attracted widespread attention as a novel solution. Therefore, developing higher-quality mHealth applications and evaluating their potential to improve perinatal mental health is an important area of research.
 The purpose of this trial was to explore whether family-supported companion video sharing—a method involving the sharing of supportive videos within the family—can improve pregnant women depression and corresponding clinical indicators.

1. **Research Profile：**

The study was designed as an intervention randomized controlled trial to investigate whether a mobile health intervention could improve perinatal depression and related clinical indicators in pregnant women. It also compared the difference in effectiveness between the mobile health intervention model and the traditional management model in this population. The aim was to provide a basis for improving maternal depressive symptoms or tendencies and enhancing the quality of life of pregnant women during the perinatal period. The study will explore a scalable and sustainable health education intervention model for perinatal depression patients using mobile smart devices. The expected number of participants is 40 pregnant women. Participants will be randomly assigned to one of two groups: the Companion Video-Sharing Group or the traditional care group. The entire assignment process is randomized.

During the first six weeks after enrollment, the Companion Video-Sharing Group will film one companion video weekly, each corresponding to one of six thematic topics, under the guidance of obstetric nurses. The recorded videos will be sent to the research group's WeChat account. At the end of each week during the first six weeks, obstetricians, labor-and-delivery nurses, and the research support team will meet with participants via WeChat video calls to address any clinical questions and care issues that you and your Companion have encountered during the week.

For pregnant women in the Traditional nursing group, at the end of each week for the first six weeks after enrollment, the obstetricians and obstetric nurses, as well as the research assistants, organized a WeChat online video exchange, where the pregnant women and her Companion could consult with the research group on perinatal-related clinical problems encountered during the week as well as on nursing care, and one-on-one replies were provided by the obstetricians and obstetric nurses.

You will be followed up by the researchers at regular intervals, four times at 15 weeks of gestation, 18 weeks of gestation, 36 weeks of gestation, and 4 weeks after delivery. All patients will receive all necessary diagnostic or therapeutic procedures, as well as due health literacy education.

1. **Inclusion criteria**

(1) gestational age of 12 weeks (within a one-week window); (2) age of 18 years or older; (3) absence of plans for pregnancy termination; (4) commitment to reside in Shandong for at least one year and availability for study follow-up (with all visits conducted at Dongying People's Hospital until delivery); (5) EPDS-Dep-5 score of ≥ 4 during depression screening; (6) consistent presence of a designated companion throughout pregnancy; and (7) comprehensive understanding and voluntary participation in the trial by both pregnant women and their companions (informed consent)

1. **Exclusion criteria**

(1) lacking a smartphone or being unfamiliar with WeChat; (2) receiving ongoing psychiatric treatment or using psychotropic medications; (3) having significant organ dysfunction that impairs daily functioning; (4) participating concurrently in a similar depressive intervention clinical study; and (5) refusing to participate in the study.

1. **Responsibilities of subjects**

As a subject participating in a research study, you have the following responsibilities: to provide truthful information about your medical history and current physical condition; to inform the research physician about any problems you have had during this trial; to avoid taking restricted medications and foods; and to inform the research physician if you have been or are currently involved in any other research studies.

1. **Possible benefits of participation in the study**

1.The benefits of the research for you personally

This study may prevent you from becoming depressed or improve your mental health during the perinatal period, but we cannot guarantee this. The health education knowledge in this study is important to help you understand your own mental health, and although it cannot directly prevent depression, it can help you answer questions about various illnesses and continue to benefit from it.

2.Possible benefits of research for social groups

Although participation in this trial may not provide you with direct benefits, you will receive specialized pregnancy knowledge from professional obstetricians and nurses at Dongying People's Hospital during your participation.

1. **Possible adverse effects, risks and discomforts of participating in the study**

There is no financial loss to you as a result of this trial, but the questionnaire will take a small amount of your time, some of the questions may cause you discomfort and there may be a risk of disclosure of your privacy, and there may be certain risks that the researcher could not have foreseen. There are no adverse reactions to this trial. This trial will treat the information you fill in as absolutely confidential, and there are strict review criteria for data management.

1. **Related treatment and compensation**

If you experience any discomfort or other reaction during this period, please inform your doctor, who will take prompt action as appropriate. This trial will not affect your treatment regimen and is a health education study only, which will not have any adverse effect on your access to regular treatment.

1. **Voluntary option to participate and withdraw from the study**

Your participation in this trial is completely voluntary. If you volunteer and are successfully enrolled, we hope that you will continue to complete the trial; you may decide at any time, for any reason, not to participate in the trial or to withdraw from the trial, and at the time of withdrawal you will be assessed for any abnormalities and will continue to be followed up until you have returned to normal or have reached a stable stage.

1. **Confidentiality of personal information**

If you decide to participate in this trial, your participation in the trial and your personal information during the trial will be kept confidential. The physician in charge of the study and other researchers will use your medical information to conduct the study. This information may include your name, address, telephone number, medical history and information obtained during your research visit. Your file will be kept in a locked file cabinet and will be accessible only to the researchers. No information about you personally will be disclosed when the results of this study are published.

1. **How to get more information?**

You can keep up to date with information about this trial and the progress of the study. If you have questions about this trial, or if you experience any discomfort or injury during the course of the study, or if you have questions about the rights and interests of the participants in this study, you can contact a member of the research team.

Before you (or your legal representative) decide to enroll in this clinical trial/research study, please read this Informed Consent Form carefully, and the research physician will help answer your questions about the product being tested and about this trial/research study. If you are participating voluntarily, after reading this information, please sign your name and date the last page of the informed consent form.

**Part II. Signature of informed consent**

1.I have carefully read the instructions in the Informed Consent Form for this research project, the research physician has thoroughly explained it to me and answered my questions about it, and I am fully aware of the purpose and process of participating in this trial/research study and my rights and risks. I am voluntarily participating in this trial/study and agree to cooperate with the research physician in the treatment and follow-up as outlined in the informed consent form and to do my best to complete this trial/study.

Subjects sign here：

*（printed letters*） （handwriting） date

Or the subject's legal representative signs here (if necessary):

*（printed letters）* The relationship with you is：

（handwriting） date

2. I or my researchers have fully explained and justified to you the purpose of the trial/study, the procedures, and the possible risks and potential benefits of your participation in the trial/study, and have answered all of your questions about it satisfactorily。

The investigator or the informing physician designated by the investigator signs here:

*（printed letters）* （handwriting） date

You and the researcher will both sign two identical copies of the informed consent form and each will keep one copy.
